# Formation of a traditional Chinese medicine self-assembly nanostrategy and its application in cancer: a promising treatment

**DOI:** 10.1186/s13020-023-00764-2

**Published:** 2023-06-06

**Authors:** Ju Huang, Yu Zhu, Hang Xiao, Jingwen Liu, Songtao Li, Qiao Zheng, Jianyuan Tang, Xiangrui Meng

**Affiliations:** 1 Hospital of Chengdu University of Traditional Chinese Medicine, Chengdu, People’s Republic of China; 2 Capital Medical University, Beijing, People’s Republic of China; 3 TCM Regulating Metabolic Diseases Key Laboratory of Sichuan Province, Hospital of Chengdu University of Traditional Chinese Medicine, Chengdu, People’s Republic of China

**Keywords:** Traditional Chinese medicine, Self-assembly nanostrategy, Cancer, Formation, Application, Combined chemotherapy

## Abstract

Traditional Chinese medicine (TCM) has been used for centuries to prevent and treat a variety of illnesses, and its popularity is increasing worldwide. However, the clinical applications of natural active components in TCM are hindered by the poor solubility and low bioavailability of these compounds. To address these issues, Chinese medicine self-assembly nanostrategy (CSAN) is being developed. Many active components of TCM possess self-assembly properties, allowing them to form nanoparticles (NPs) through various noncovalent forces. Self-assembled NPs (SANs) are also present in TCM decoctions, and they are closely linked to the therapeutic effects of these remedies. SAN is gaining popularity in the nano research field due to its simplicity, eco-friendliness, and enhanced biodegradability and biocompatibility compared to traditional nano preparation methods. The self-assembly of active ingredients from TCM that exhibit antitumour effects or are combined with other antitumour drugs has generated considerable interest in the field of cancer therapeutics. This paper provides a review of the principles and forms of CSAN, as well as an overview of recent reports on TCM that can be used for self-assembly. Additionally, the application of CSAN in various cancer diseases is summarized, and finally, a concluding summary and thoughts are proposed. We strongly believe that CSAN has the potential to offer fresh strategies and perspectives for the modernization of TCM.

## Introduction

Traditional Chinese medicine (TCM) is used to prevent and cure a wide range of illnesses [1–4], and it includes a variety of plant-, animal-, and mineral-based medicinal substances. Since active components in TCM exhibits low bioavailability, novel formulations and administration methods are needed to enhance the efficiency and safety of drug delivery systems. Nanotechnology is used to reduce TCM-related issues such as poor solubility, lack of targeting, nonspecific distribution, high systemic toxicity, and a short half-life. Conventional nanomaterials, on the other hand, produce low yields, are expensive to prepare, accumulate in vivo, and are not degradable; thus, it is difficult to use conventional materials significantly in clinical applications [5–9].

Compared to traditional nanoformulations, self-assembled nanoformulations are easier to manufacture, more efficient and exhibit higher biodegradability and biocompatibility. Active components of TCM that possess self-assembly capabilities include alkaloids, organic acids, flavonoids, terpenoids, polysaccharides, and proteins derived from natural plants. By utilizing various types of noncovalent bonds, including intermolecular hydrogen bonds, van der Waals forces, π-π stacking, electrostatic interactions, and coordination bonds, it is feasible to construct stable self-assembly systems with predetermined architectures [10, 11] (such as NPs, micelles, fibres, nanotubes, and vesicles). CSAN refers to a process that utilizes TCM to form self-assembled NPs (SAN) with particle sizes ranging from 10 to 1000 nm in this paper. CSAN can be prepared using single or combination TCM that can self-assemble to produce pure natural carrier-free nanoformulations. CSAN can improve the solubility and in vivo bioavailability of insoluble TCM active components. Since CSAN may increase the solubility and bioavailability of insoluble TCM active components, it may also be employed as a carrier-free nanodelivery system for drug delivery and therapeutic treatment. CSAN is effective, secure, straightforward, inexpensive, and does not require any specialized equipment. Additionally, it provides more benefits than conventional nanoformulations and is favourable for the clinical translation of nanomedicines.

CSAN has been shown to exert an antitumour impact through the active components found in TCM [12]. Furthermore, it has been found to exhibit a synergistic effect when used in conjunction with other antitumour drugs [13]. Moreover, CSAN shows potential as a natural nanocarrier for antitumour drugs [14]. It can also be utilized to construct nanopreparations with metal ions via coordination bonds for photothermal treatment or tumour imaging [15], which could be applied for the diagnosis and treatment of malignant tumours [16]. Therefore, we provide an overview of the self-assembly mechanism, composition, and use of CSAN in tumour disorders, and the integration of active components of TCM with self-assembly technology is expected to broaden the scope of TCM modernization.

## The mechanism of CSAN

**Fig. 1:**
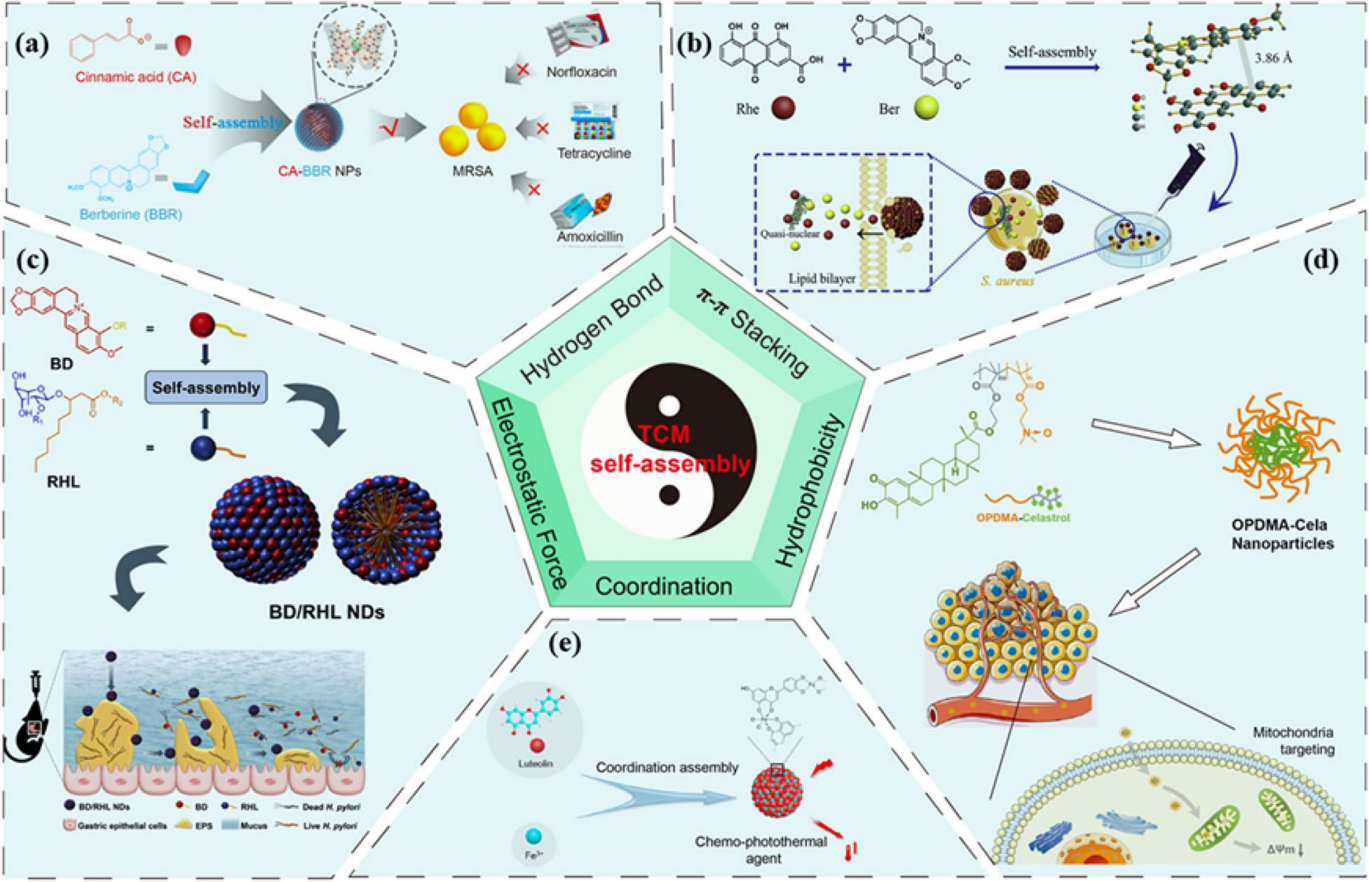
Different self-assembly forces in CSAN. **a** CA-BBR NPs formed by hydrogen bonding which as the main self-assembly force. Copyright 2020 American Chemical Society. **b** Rhe-BBR NPs formed by π-π stacking which as the main self-assembly force. Copyright 2018 Elsevier. **c** BD/RHL NDs formed by electrostatic force which as the main self-assembly force. Copyright 2020 Elsevier. **d** OPDMA-Cela NPs formed by hydrophobic interaction which as the main self-assembly force. Copyright 2022 Elsevier. **e** Lut/Fe3 + NPs formed by coordination which as the main self-assembly force. Copyright 2020 Elsevier

TCM is composed of a complex and diverse set of active components, such as alkaloids, organic acids, flavonoids, terpenoids, polysaccharides, and proteins. These components possess functional groups and multiple action sites, which make it easier for them to form noncovalent bonds. Additionally, their unique structure and complicated physical and chemical characteristics facilitate self-assembly. The active components of TCM primarily self-assemble through noncovalent interactions, including hydrogen bonds [17], van der Waals forces, π-π stacking [18], hydrophobic interactions [19], electrostatic interactions [20], and coordination interactions [15] (Fig. 1). The self-assembly process does not simply result from weak interactions between countless atoms, ions, and molecules. Rather, a diverse range of particles spontaneously come together to form a tightly organized structure, resulting in an overall complex synergy. In the process of self-assembly, it is uncommon for a single noncovalent bond to be the sole force. Rather, multiple noncovalent bonds often work together in tandem to aid the process. Therefore, determining the mechanism of CSAN is imperative. In this paper, we provide a detailed account of the key examples in CSAN (Table 1) and classify them according to the variations in their self-assembly driving force.

**Table 1:** Common self-assembly forces in CSAN

Self-assembly force	Self-assembly mechanism	Active ingredient	Primary source	Structure	Refs.
Hydrogen bond	Hydrogen bond	OA	Glossy privet fruit		[19]
		PTX	Taxus chinensis		
	Hydrogen bond and Hydrophobic interaction	OA	Glossy privet fruit		[29]
		GA	Licorice		
	Hydrogen bond and π-π stacking	BBR	Coptis chinensis		[17]
		CA	Cinnamon bark		
	Hydrogen bond and van der Waals forces	Sito	Arisaema heterophyllum Blume		[28]
Hydrophobic interaction	Hydrophobic interaction	GW	Daphne genkwa		[38]
		GA	Licorice		
	Hydrophobic interaction and hydrogen bond	UA	Loquat leaf		[12]
	Hydrophobic interaction and π-π stacking	Cela	Tripterygium wilfordii		[41]
	Hydrophobic interaction and electrostatic interaction	WOG	Baikal skullcap		[20]
		BBR	Coptis chinensis		
Electrostatic interaction	Electrostatic interaction and hydrophobic interaction	BA	Baikal skullcap		[20]
		BBR	Coptis chinensis		
π-π stacking	π-π stacking and hydrogen bond	Rhe	Rheum officinale		[18]
	π-π stacking, van der Waals forces, and hydrogen bond	LDA	Liquidambar formosana		[14]
Coordination interaction	Coordination interaction	Lut	Lonicera japonica Thunb		[15]

### Hydrogen bonding force

Hydrogen bonds are among the primarily weak electrostatic forces between atoms in nature and is responsible for the shape and structure of substances as well as their physical and chemical properties [21–23]. The bonds possesses intermediate strength, directionality, and saturation and is an excellent driving factor for the formation and stabilization of supramolecular self-assembly systems. Hydrogen bonds, therefore, play an important role in the formation of CSAN. When molecules are joined together by hydrogen bonds, single and multiple hydrogen bonds are formed. The greater the strength of the multiple hydrogen bond, the greater the binding energy and stability between molecules. Since hydrogen bonds play such an important role in the process of self-assembly, most published research on the self-assembly of TCM is based on hydrogen bonds.

Berberine (BBR), a quaternary ammonium alkaloid extracted from the medicinal plant *Coptis chinensis* (Ranunculaceae), is widely used in TCM for its antibacterial and anti-inflammatory properties [24, 25]. It is easy to form nanoformulations due to its polyaromatic ring structure and quaternary ammonium ions. Many studies have found that BBR can self-assemble with various active components of TCM. Huang et al. [17] found that the combination of BBR and cinnamic acid (CA) can create a stable SAN through the interaction of hydrogen bonds. This interaction leads to the formation of butterfly shaped one-dimensional self-assembly units, as evidenced by single crystal X-ray diffraction. The π-π stacking interaction promoted the formation of a layered three-dimensional crystal with a stacking spatial configuration and eventually completed the self-assembly process. Among these, the carbonyl group of CA may form a hydrogen bond with the nitrogen atom of BBR, and aromatic ring association was a major source of the stacking structure. This self-assembly approach improved drug bioavailability and might be a viable way to combat bacterial resistance. Tian et al. [26] found the self-assembly mechanism of rhein (Rhe) and BBR by using nuclear magnetic resonance spectroscopy and X-ray single-crystal diffraction. Rhe, originating from TCM *Rheum palmatum* (Polygonaceae), serves as the layered stacked backbone of Rhe-BBR NPs, which are stabilized by hydrogen bonds. Additionally, BBR was embedded in the structure through π-π stacking and electrostatic interactions, further enhancing the stability of the self-assembly. This self-assembly approach improved the bioavailability of the drugs and exhibited a better antibacterial effect than that of the single drug group. By hydrogen bonding and π-π stacking, BBR and 3,4,5-methoxyic acid (3,4,5-Tca) self-assembled to create phytochemical NPs with high antibacterial activity [27]. B-Sitosterol (Sito), which is derived from *Arisaema heterophyllum Blume* (Araceae), has the ability to self-assemble and form natural product gelators [28]. The self-assembly process was initiated by hydrogen bonds and was further strengthened by van der Waals forces. The resulting dimers were then organized longitudinally to create a secondary structure. The secondary structure continued to build up, resulting in fibrous network aggregates. This self-assembly approach improved drug stability and improved cancer cell inhibition and drug delivery. In another study, oleanolic acid (OA) and glycyrrhetinic acid (GA) formed a self-assembly [29] through hydrogen bonding and hydrophobic interactions. Because the hydrophilic surface of OA tended to match that of GA (hydrogen bond donors and acceptors), the hydrophilic hydrogen-bond connectivity at the surface increased, and hydrogen-bond interactions developed. This self-assembly approach improved the biological solubility and stability of OA and GA, resulting in better antitumour effects. Both OA and irsolic acid (UA) are triterpenoids that can self-assemble with PTX to form NPs. According to Wang et al. [19], the major force of OA-PTX NPs was hydrogen bonding, whereas the main force of UA-PTX NPs was hydrophobic interactions. In the molecular modelling process, OA-PTX NPs possessed more and stronger hydrogen bonds, which might explain why OA-PTX NPs were more stable. This self-assembly approach improved the solubility and antitumour activity of the three drugs.

Hydrogen bonding plays a crucial role in the self-assembly of TCM. The self-assembly of TCM through hydrogen bonding offers several advantages, such as increased solubility, improved stability of TCM components, and enhanced absorption of medicine.

### Hydrophobic interaction

Hydrophobic interactions occur when hydrophobic groups in aqueous solution cluster close to each other to avoid water, which is common in nature [30–32]. In many instances of self-assembly, hydrophobic contact is the driving force, and hydrogen bonding is created simultaneously. There may be a fine balance between hydrophobic contacts and hydrogen bonding forces [33, 34].

Many triterpenoids have been reported to generate self-assembled NPs via hydrophobic interactions. UA is a pentacyclic triterpene produced from *Eriobotrya japonica* (Rosaceae), *Salvia officinalis* (Lamiaceae), *Fructus Chaenomelis Lagenariae* (Caricaceae), and other TCMs [35–37]. Hydrophobic interactions and hydrogen bonding between UA molecules resulted in the formation of UA NPs [12]. Furthermore, the intensity of hydrophobic interactions and hydrogen bonds can influence NP stability. The hydrophobic interaction was primarily responsible for the self-assembly of UA- paclitaxel (PTX) NPs [19]. The benzene ring in the structures of UA and PTX had a strong hydrophobic interaction, which caused the two methyl groups in UA to generate a strong hydrophobic effect with the two benzene rings coupled with the amide bond in the structure of PTX. The mechanism of NPs prepared with yuanhuacine and glycyrrhizic acid that originate from *Genkwa Flos* (Thymelaeaceae) and *Glycyrrhiza glabra* (Fabaceae) has been investigated [38]. 1H-NMR and NOESY 2D NMR spectra confirmed that H-14 and H-20 of Yuanhuacine and Glu groups of glycyrrhizic acid were self-assembly binding sites, and the self-assembly process was driven by hydrophobic interactions. The interaction was inwardly configured with hydrophobic pentacyclic triterpenoids from glycyrrhizic acid and yuanhuacine and outwards configured with hydrophilic glucuronic acid. BBR and PTX were linked by disulfide bonds. Cheng et al. [13] discovered that the conjugate molecule could self-assemble in an aqueous solution through hydrophobic interactions and π-π stacking, resulting in the formation of PTX-ss-BBR NPs. This was determined using molecular dynamics simulations. PTX molecules with numerous phenyl groups were surrounded by BBR inside the nanostructure, eventually forming a stable nanostructure. Celastrol (Cela) is a triterpenoid metabolite derived from *Tripterygium wilfordii* (Celastraceae) [39, 40]. In an aqueous solution, Cela may self-assemble into NPs [41] with hydrophobic and π-π stacking interactions. This was due to the amphiphilic character of Cela and the presence of a significant number of aromatic rings in the inner core.

### Electrostatic force

Electrostatic interactions are defined as an electrostatic attraction between charged groups, dipoles, and induced dipoles. A dipole distance occurs between polar molecules, and dipole molecules interact electrostatically. This intermolecular contact is known as electrostatic force, and it occurs during self-assembly [33, 42].

Baicalin (BA) and wogonoside (WOG) are flavonoids that are major components of *Scutellariae* radix (Lamiaceae) [43, 44]. In aqueous solution, BA and BBR self-assembled into NPs [20] via the electrostatic interaction generated between the carboxyl groups in BA and the quaternary ammonium ions in BBR. The electrostatic interaction promoted the formation of one-dimensional complex units, whereas the hydrophobic interaction facilitated further three-dimensional self-assembly, resulting in homogeneous spherical BA-BBR NPs. WOG and BA may self-assemble to produce nanofibers (NFs) in an aqueous solution, in which the major force is a strong hydrophobic effect. This was because WOG-BBR is not amphiphilic and hence cannot self-assemble into NPs but rather precipitates in aqueous solution due to its high hydrophobicity. As a result, type I planar hydrophobic molecules are more prone to form NFs. Importantly, there are significant differences in antibacterial effectiveness between the two, with the NPs significantly surpassing the NFs. BBR derivatives (BDS) self-assemble with rhamnolipids (RHL), an anionic surfactant secreted by *Pseudomonas aeruginosa*, to form stable nanodrugs (BD/RHL NDS) [45], and molecular dynamics modelling confirms that the formed self-assembly was due to electrostatic and hydrophobic interactions. Electrostatic interactions require attraction, repulsion, or directionality between particles and may occasionally affect the size and length of nanostructures [42].

### π-π stacking

π-π stacking is a spatial organization of aromatic compounds that involves a mild interaction between aromatic rings. It generally occurs between two molecules that are substantially electron-rich and electron-poor. It is a noncovalent bond interaction with equal important as hydrogen bonding, and its mechanism is more complex than that of hydrogen bonding [46, 47].

Zheng et al. [18] used spectroscopy and single crystal X-ray diffraction to investigate the mechanism of direct self-assembly of hydrogels between Rhe molecules extracted from the TCM *Rheum palmatum* (Polygonaceae) and observed that certain Rhe molecules were deprotonated to form Rhe sodium when the pH was between 8.0 and 9.4. Through π-π stacking and hydrogen bonding, the Rhe monomer and sodium Rhe formed J-type aggregates, which then combined to create dimers. These dimers then assembled into trimers, tetramers, and other higher-order aggregates. Ultimately, this process resulted in the formation of a Rhe self-assembling gel. The study revealed that carboxyl groups played a crucial role in the gelation process, as emodin, chrysophanol, aloe-emodin, and emodin A failed to produce hydrogels. Additionally, liquidambaric acid (LDA) is a triterpenoid that originates from *Liquidambar formosana* (Altingiaceae). Its molecular structure can be divided into the following main parts: the alkane polycyclic skeleton (tricyclic, tetracyclic, and pentacyclic), the alkene (double bond), and the oxygen-containing moiety (such as hydroxyl). Zhi et al. [14] examined the potential interaction mechanism of these different parts through 2D NMR and 1D 1H NMR and found that LDA NPG initially developed one-dimensional fibres through the self-assembly of the following distinct noncovalent interactions: π-π stacking, van der Waals forces, and hydrogen bonding. These fibres then interacted with the solvent, resulting in the formation of NPG with a three-dimensional network fibre structure over time. We observed that TCMs with aromatic ring conjugated structures tended to self-assemble directly under π-π stacking or π-π stacking coordination.

### Coordination

Coordination is a medium-strength intermolecular force that is weaker than covalent interactions and falls between hydrogen bonds and van der Waals interactions in terms of strength. The intensity of coordination is influenced by external factors [48, 49].

Luteolin (Lut) is a natural flavonoid that can be found in *Lonicera japonica* (Caprifoliaceae) [50, 51]. Recent research has shown that Lut can self-assemble with Fe^3+^ to form NPs [15]. This coordinated assembly with iron ions resulted in supramolecular photothermal action, enabling the NPs to act as chemotherapeutic or photothermal agents. It is worth mentioning that various catechol flavonoids, such as Lut, quercetin, kaferol, baicalin, and catechin, can combine with metal ions to self-assemble and form NPs. This coordination produced supramolecular photothermal action, allowing the NPs to function as chemotherapeutic or photothermal agents.

Many active components in TCM can self-assemble due to their unique structures. As a result, these components exhibit an ability to form nanoformulations with stable nanostructures, which is attributed to noncovalent processes, including hydrogen bonding, π-π stacking, electrostatic interactions, hydrophobic interactions, and coordination.

## The self-assembly forms of CSAN

An increasing number of studies on the self-assembly of TCM [52–54] have been reported, and these studies range from the active components of TCM monomers to decoctions of single or compound TCM. In this paper, we classify and summarize CSAN based on its different forms of self-assembly.

### Pure natural nanoformulations formed by self-assembly of single or compound TCM components

**Fig. 2:**
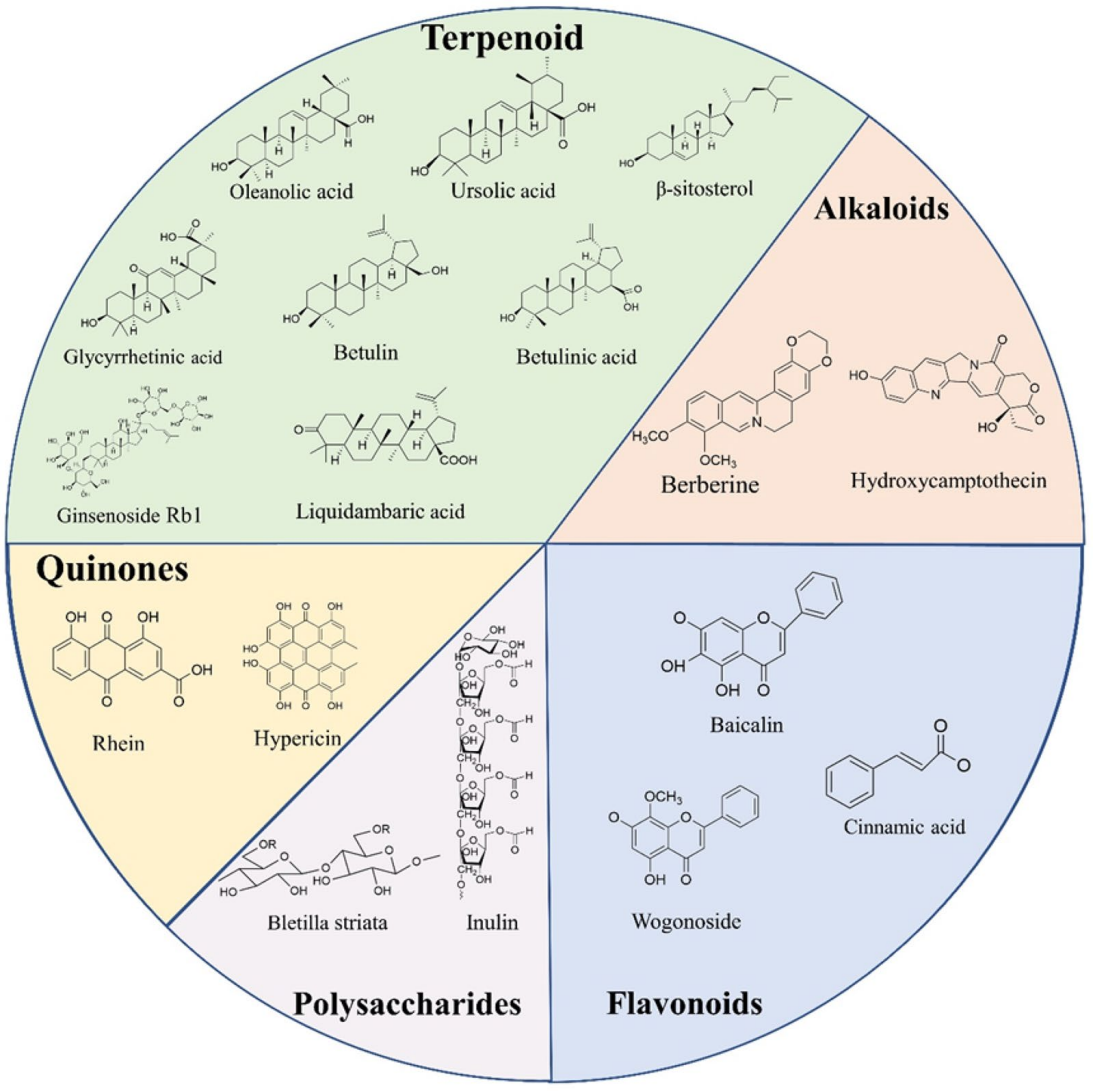
Chemical structure of TCM with self-assembly ability

Nanoformulations produced by the self-assembly of single or compound TCMs can increase the solubility and bioavailability of TCM active components and can be employed as natural nanodelivery carriers, which can play a coordinating role while exerting their own therapeutic effect. Due to the distinctive three-dimensional structure, multifunctional groups, and good biocompatibility of TCM, it is easy to build great nanostructures (Fig. 2), amplifying the benefits of TCM. Furthermore, compared to the present nanopreparation procedure, this method is simpler, more ecologically friendly, and safer. Because of these properties, this technique has garnered widespread attention.

#### Self-assembly of TCM with the same components

##### Terpenoids

Terpenoids, which utilize isoprene as a basic structural unit, are among the most abundant natural phytochemicals [55–57]. Terpenoids exhibit a distinct rigid skeleton, chiral centre, and many modification sites, as well as varied amounts of hydroxyl and carboxyl groups, and fold easily in diverse mediums to produce self-assembled NPs. Terpenoids are now the focus of nanoself-assembly research. UA is a pentacyclic triterpenoid found in a variety of TCMs. According to the findings of Braja Gopal Bag's study [58], UA molecules self-assembled in most organic and aqueous organic solvents to form nanostructures, such as vesicles, tubes, fibres, flowers, and supramolecular gels, which were employed to entrap the anticancer drug doxorubicin (DOX). Fan et al. [12] prepared UA NPs by using the solvent exchange method, which was straightforward and efficient, and carriers or water-soluble medicines were not needed throughout the procedure; as a result, greater drug release ability and anticancer effects were obtained than those of free UA. GA, a pentacyclic triterpene, is among the most important active components in *Glycyrrhiza glabra* [59, 60]. GA may self-assemble in aqueous solution, form complexes with other drugs, improve the solubilization of poorly soluble drugs, and be utilized as DSS to deliver drugs and improve their solubility and bioavailability [61]. In in vivo studies, Yang et al. [62] applied GA micelles to deliver PTX in vivo, which enhanced oral bioavailability by nearly sixfold compared to that of free PTX. Wang et al.[63] used GA micelles to deliver podophyllotoxin (POD) transdermally, increasing POD distribution in the epidermis while decreasing skin inflammation. Paeoniflorin (Pae), a water-soluble monoterpene glycoside, exhibits high therapeutic value in autoimmune and inflammatory illnesses [64, 65]. Shen et al. [61] used GA micelles to enhance Pae oral absorption. Pae-loaded GA micelles increased Pae release and intestinal permeability compared to that of free Pae. LDA is a pentacyclic triterpene derivative extracted from the plant *Liquidambar formosana* (Altingiaceae). Zhi et al. [14] formed LDA natural product gels (NPGs) with good self-healing properties, controlled gelation, safety, and prolonged release through self-assembly of the solvent method. Sito is a tetracyclic triterpenoid derived from the plant *Arisaema heterophyllum Blume* (Araceae). He et al. [28] produced Sito NPG using heat-cool and ultrasonic technologies. In vitro, Sito NPG demonstrated equivalent or better carcinogenesis than that of Sito NPG and showed potential as a natural delivery vector. Braja Gopal Bag et al. [66] discovered self-assembly reactions for several triterpenoids in diverse liquids, including betulinic acid, OA, GA, gingerfruit acid, and its derivatives. These self-assembling reactions might be used to encapsulate fluorophores, DOX, and other molecules. Terpenoids have been extensively explored in CSAN owing to their ease of self-assembly.

##### Proteins

Proteins possess a distinctive structure that enables them to self-assemble via noncovalent interactions. Recently, there has been a growing interest in researching plant protein NPs obtained from TCM, such as licorice protein [67], almond protein [68], heterophylla protein [69], and several others. Protein self-assembly is a common occurrence in nature. Zhou et al. [67] identified glycated licorice protein (GLP) from licorice (*Glycyrrhiza uralensis* Fisch.). This protein is novel, plays an unknown biological function and exhibits a molecular weight of 28 kDa. The team created GLP NPs through decocting, and these NPs demonstrated remarkable pH response properties. Furthermore, they were found to promote the growth of normal liver cells and dissolve insoluble astragaloside IV. Additionally, Radix Pseudostellariae (Caryophyllaceae, *Pseudostellaria heterophyll*) protein (RPP) displayed significant self-assembly properties and antioxidant activities. The combination of RPP with curcumin (Cur) has resulted in the development of RPP NPs loaded with Cur, forming nanocomplexes that exhibit enhanced photostability, antioxidant activity, and cell uptake efficiency [69]. This advancement should enable the delivery of more functional bioactive components. NPs were prepared using RPP-polysaccharide (CP3) conjugates through a heat treatment condensation technique [70], and these NPs can rapidly release DOX at an acidic pH. Additionally, protein NPs are present in the aqueous solution of Coptis chinensis extract [71], which can serve as a natural drug carrier to enhance the plasma exposure and biological activity of BBR. Apricot Kernels, commonly known as *Prunus armeniaca* Linne var. *ansu* Maximowicz, is widely used as a TCM [72, 73]. *Semen Armeniacae Amarum* Protein (SAP) found in the decoction can self-assemble to form SAP NPs [68]. SAP NPs are loaded with the anticancer drug PTX to form PTX-SAP-NPs, which compared to fee PTX, were more effective against tumours. Huang et al. [74] discovered ivy NPs (INPs) from the adventitious roots of English ivy (Araliaceae; *Hedera helix*). These INPs can create INP-DOX nanoconjugates by encapsulating DOX through electrostatic and hydrophobic interactions and show promise in the treatment of tumours. Zhou et al. [75] isolated Ban-Lan-Gen (BLG) NPs from the decoction of *Isatis indigotica* Fort. They identified the main components of BLG NPs as two types of glycosylated proteins and found that they exhibit a tumour-inhibitory function. Protein NPs derived from TCM exhibit favourable pharmacological properties and exceptional bioavailability; thus, they are a highly promising avenue for developing therapeutic nanocarriers.

##### Polysaccharides

Polysaccharides are natural macromolecules generated by the chemical polymerization of several monosaccharides [76, 77]. They are abundant in nature and exhibit antitumour [78], antioxidant [79], and other qualities [80]. The skeletal structure of polysaccharides is densely packed with hydrophilic groups. The balance between hydrophilic and hydrophobic groups can be achieved by introducing hydrophobic aromatic groups or alkyl chains to polysaccharide molecules, which promotes polysaccharide self-assembly. Stearic acid (SA)-modified *Bletilla striata* polysaccharides (BSPs-SA) isolated from the stem tubers of *Bletilla striata* (Orchidaceae) could be self-assembled to form BSPs-SA NPs [81]. It displays an antitumour effect after loading with DOX. Angelica polysaccharide (ASP), a plant polysaccharide extracted from *Angelica sinensis* radix (Apiaceae), shows increased biocompatibility, is water soluble, and has the capacity to target the liver internally [82, 83]. When ASP is modified with a hydrophobic group (deoxycholic acid), it can self-assemble to generate ASP-DOCA NPs [84]. After ASP-DOCA NPs were encapsulated with DOX, they demonstrated a greater antitumour effect than that of free DOX. Inulin is a polysaccharide derived from *Cichorium intybus* radix (Asteraceae). Carboxymethyl-modified inulin may self-assemble into spherical NPs [85], which can be employed to treat spinal cord injury collaboratively. Inulin with a high molecular weight synthesized by enzymes can form stable spherical nanoparticles with an average diameter of 112 ± 5 nm as a drug delivery system [86]. Therefore, polysaccharides are attracting the attention of researchers due to their excellent biocompatibility and self-assembly properties.

##### Quinones

Quinones tend to form nanostructures due to their polycyclic rings and hydrogen bonds [87]. Rhe is a lipophilic anthraquinone molecule found in *Rheum palmatum* (Polygonaceae) that contains hydrophobic planar anthraquinone units as well as numerous hydroxyl and carboxyl groups that easily self-assemble. Rhe may self-assemble into Rhe hydrogel [18] without any structural alteration, providing long-lasting release as well as increased anti-neuroinflammatory effects by downregulating pro-inflammatory cytokines and neurotoxic factors. Hypericin, a dianthraquinone molecule, is extracted from *Hypericum perforatum* (Hypericaceae) and is among the strongest photosensitizers in nature [88, 89]. It specifically targets necrotic tumour lesion sites and may self-assemble with demyoglobin to produce nanostructures [90], which increase hypericin bioavailability, fluorescence, and bacterial photoactivation.

##### Others

Ginger, the rhizome of *Zingiber officinale* (Zingiberaceae), is among the most widely used natural products [91]. Ginger NPs can be employed as natural nanocarriers, which offer benefits over conventionally manufactured nanocarriers. Zhang et al. [92] isolated a significant number of NPs from ginger, and these ginger-derived nanovectors (GDNVs) demonstrated good stability and drug release, making them suitable to deliver DOX. NPs derived from edible ginger (GDNPs 2) [93] contain a high concentration of lipids, a minor quantity of protein, ~ 125 microRNAs (miRNAs), and a high concentration of bioactive ginger components (6-gingerol and 6-gingerol). GDNPs 2 targets inflamed intestinal mucosa to prevent chronic colitis and colitis-related malignancies. NPs composed of edible ginger-derived lipids, called ginger-derived lipid vectors (GDLVs) [94], were loaded with anti-CD98 siRNA to form siRNA-CD 98/GDLVs. At extremely low dosages, oral SiRNA-CD 98/GDLVs suppressed colon CD98 gene expression specifically and efficiently. *Rabdosia rubescens* (Lamiaceae) is commonly used as an antithrombotic medicine. Peng et al. identified five nucleotides, four diterpenoids, and 21 phenolic acids from the aqueous extract of *Rabdosia rubescens* leaves (AERL), and AERL can form NPs in a concentration-dependent manner in water [95]. In addition, AERL concentration-dependently inhibited platelet aggregation in vitro and dose-dependently inhibited thrombosis in vivo, and it was predicted that frosmarinic acid may be its main antithrombotic active component.

#### Self-assembly of TCM with different components

##### Alkaloids and flavonoids

BBR, an isoquinoline alkaloid, is the main active component of *Coptis chinensis F.* (Ranunculaceae) and is widely used in clinics. However, BBR exhibits a low water solubility and an unpleasant bitter flavour [25, 96]. These flaws can be effectively improved by CSAN. Simultaneously, the quaternary ammonium ion and benzene ring structure of BBR can be joined with other natural products, particularly flavonoids, to form NPs with suitable particle sizes. Because of its strong self-assembly ability and antibacterial potential, BBR has become among the most investigated drugs in TCM self-assembly. BA and WOG are flavonoids that are major components of *Scutellariae* radix (Lamiaceae). In an aqueous solution, BBR and BA self-assemble to generate stable BBR-BA NPs [20], which exhibit high biocompatibility and greatly increased antibacterial and biofilm removal properties. BBR and WOG can also self-assemble to form BBR-WOG NPs. Notably, BBR-WOG NPs exhibit much lower antibacterial activity than that of BBR-BA NPs. Alkaloid components easily interact with organic acid components to self-assemble. In aqueous solution, BBR and CA may self-assemble to generate CA-BBR NPs [17] without any carriers or additives during the self-assembly process. CA-BBR NPs are more effective than various first-line drugs at inhibiting multidrug-resistant Staphylococcus aureus (MRSA). BBR and 3,4,5-TCa could self-assemble in aqueous solution to create BCR-3,4,5-TCa NPs [27], which outperformed first-line amoxicillin, norfloxacin, and their self-assembled predecessors against MRSA.

##### Alkaloids and quinones

In an aqueous solution, BBR and Rhe may self-assemble to form BBR-Rhe NPs [26]. that can bind to bacterial surfaces, increase antibacterial activity against Staphylococcus aureus and exhibit a higher biofilm clearance rate and synergistic antibacterial ability than that of BBR or Rhe. Currently, the self-assembly technique of BBR-related TCM is mostly applied in antibacterial studies, and it has demonstrated strong antibacterial efficacy.

##### Alkaloids and protein

In clinical practice, the combination of *Glycyrrhiza uralensis* with *Aconiti Kusnezoffii Radix* can effectively remove the toxicity of *Aconiti Kusnezoffii Radix* and improve the therapeutic effect [97]. CSAN offers a novel approach to study this phenomenon. Aconitine (AC) is an extremely poisonous alkaloid derived from several *Aconitum* plants [98]. Ke et al. [99] isolated a stable 31 kDa protein (GP) from *Radix glycyrrhizae*, and the GP-AC NPs produced by the solvent technique dramatically decreased AC toxicity.

##### Alkaloids and organic acids

Aristolochic acid (AA) is present in *Asarum* and *Aristolochia* has been reported to cause acute kidney injury (AKI) [100]. However, AA-containing herbs are very safe in combination with BBR-containing herbs in TCM, and it has been found that the supramolecular self-assembly formed by BBR and AA significantly reduced the toxicity of AA and attenuated AA-induced acute kidney injury [101]. This self-assembly strategy guided by TCM compatibility theory not only provides a better curative effect but can also explain the TCM compatibility theory.

##### Terpenoids

OA and GA are triterpenoids from natural Chinese medicine. The antitumour effect of OA-GA NPs [29] was much greater than that of free drugs, and OA-GA NPs may be employed as a drug carrier to deliver PTX, enhance antitumour activity and minimize liver damage caused by PTX. The coassembly of components with similar pharmacological activity can improve the pharmacological efficacy of drug carriers. The *Genkwa Flos*/GA self-assembled complex was prepared by combining Genkwanine (GW, one of main ingredients from *Genkwa Flos*) with GA in an aqueous solution [38], which exhibit stronger apoptosis than that of *Genkwa Flos*. In clinical practice, researchers believes that the combination of *Genkwa Flos* and *Glycyrrhiza glabra* will enhance their toxicity. Hence, this study explored the clinical incompatibility of these two drugs from another angle.

##### Terpenoid and catechin

It has been claimed that epigallocatechin gallate (EGCG) from green functions as the "shell" of a multifunctional material coating. Zhang et al. [102] synthesized UA and EGCG. First, UA was simply self-assembled to create UA NPs, and then the surface was covered with EGCG to produce a core–shell structure. Finally, epithelial cell adhesion molecule (EpCAM) was introduced to modify UA@EGCG-Apt. The obtained UA@EGCG-Apt NPs (UEA NPs) showed synergistic tumour therapy and induced immunotherapy effects. This self-assembly approach based on the structural properties of several active components of TCM yields positive results.

##### Protein and diketones

Radix Pseudostellariae (Caryophyllaceae, *Pseudostellaria heterophyll*) and Curcuma longa *(Zingiberaceae*) are frequently utilized in illness treatment and health care. The RPP-Cur nanocomplexes exhibited better stability and reducing power than that of free Cur [103] and displayed an additive effect between Cur and RPP.

Self-assembly of TCM with different components can play a therapeutic role directly and act as a natural nanodelivery carrier, eliminating the adverse effects of loading chemotherapeutic chemicals and the added toxicity produced by long-term usage of nonbiologically active nanocarriers. The synthesis method is straightforward, efficient, and environmentally friendly, and it is being developed into a viable nanodrug delivery system.

#### Self-assembly of TCM decoctions

Decoction is among the most often utilized forms of TCM in clinical treatment [104–106]. TCM decoction is a complex dispersing system that contains solute, colloid, aggregate, emulsion, and precipitate, and many TCM decoctions can generate self-assembled NPs during decoction. The study of self-assembled NPs in TCM compound decoctions has biological significance and may provide a fresh viewpoint on the onset mechanism and safety of TCM decoctions and their active components.

Wu et al. [107] found that *Coptis chinensis* decoction (CCD) could form particle aggregates in CCD (CCD-Ps), which were primarily composed of polysaccharides, and BBR absorption in the intestinal tract could be improved by regulating tight junctions (TJs) between intestinal epithelial cells, active transport, and endocytosis. Nanometre aggregates (NAs) are present in Bai-Hu-Tang (BHT) water decoction [108], and BHT NAs are more likely to aggregate in the lung and brain and have a greater antipyretic effect than that of other BHT scattered phases. GDQ micro/nanoaggregates (MNA) included in Ge-Gen-Qin-LianTang decoction (GQD) [109] increase the absorption of BA and boost antidiabetic efficacy by dramatically decreasing the effect of oxidative damage on cell survival and function. The Ma-XingShi-Gan-Tang (MXSGT) decoction is a traditional Chinese botanical drug preparation that contains ephedra as its major component. Zhou et al. [110] isolated and characterized colloidal nanoparticles (MXSGT NPs) from MXSGT and found that MXSGT NPs are associated with most ephedrine and pseudoephedrine compounds in decoctions and can alter their biological activity. Elucidating the formation mechanism of SAN in TCM decoctions is helpful to promote research on TCM compounds.

The self-assembly of TCM decoctions is a self-assembly strategy that involves an easy preparation process and enhanced performance. Sometimes self-assembly is inspired by TCM theory's drug pairing experience, such as rhubarb (Polygonaceae; *Rheum rhabarbarum*)-*Coptis chinensis*, scutellarium-*Coptis chinensis*, *Coptis chinensis*-aconite, and other famous drug pairs, and exhibits positive effects. This widely accessible, versatile, and biocompatible self-assembly method offers a fresh perspective for comprehending TCM chemicals and is likely to aid in the development of novel effective nanopreparations inspired by TCM.

### Combined self-assembly of active components of TCM and chemical drugs

The active components extracted from TCM offer several benefits, including high efficiency, low toxicity, broad effects and various targets. However, many active components in TCM, such as chemical drugs, exhibit several drawbacks, including limited solubility and low bioavailability [111–113]. Many investigations have found that active components from TCM and chemical drugs self-assemble to generate nanopreparations [114]. This self-assembly technique increases the solubility and bioavailability of the two drugs, plays a synergistic function, and reduces the negative effects of chemical drugs. The technique is currently largely employed in antitumour research.

The strategy of self-assembling drugs that exhibit combined antitumour effects into nanopreparations is particularly successful, and the effects can be applied to several antitumour pathways. PTX is a regularly used anticancer drug in clinics; however, its drawbacks, such as water insolubility and multidrug resistance, severely restrict its therapeutic use [115–117]. The self-assembly of PTX with TCM active components can enhance its solubility and lessen adverse effects, allowing for greater PTX utilization in clinics. Zou et al. [118] discovered that the natural antiangiogenic agent tetramethylpyrazine (TMP) and PTX exert a synergistic effect to inhibit the growth of ovarian cancer, so they designed PTX-ss-TMP NPs with higher cytotoxicity and stronger antiangiogenesis than that of free drugs, as well as better antitumour activity and tumour-specific accumulation. Through hydrogen bonding and hydrophobic self-assembly, UA and PTX combine to generate UA-PTX NPs [19]. The synergistic action of UA and PTX can considerably increase tumour therapy efficacy and upregulate the major antioxidant system, lowering the risk of liver harm caused by chemotherapy drugs. These self-contained bioactive nanocarriers have the potential to compensate for the inadequacies of standard nanocarriers, which offer no therapeutic or health advantages. According to research, BBR preferentially accumulates in the mitochondria of tumour cells and suppresses cancer cell growth via a different biological mechanism than that of PTX. The PTX-ss-BBR NPs [13] can target mitochondria and exhibits better antitumour effects. Gambogic acid (GBA) is the principal active ingredient in the traditional Chinese medication Garcinia Cambogia [119], and the DTX-GBA PLGA NPs [120] produced by the self-assembly approach may be used in tumour combination therapy and exhibit high potential for treating multidrug-resistant breast cancer. CSAN shows the potential to be an efficient and cost-effective drug delivery technology that improves the stability and safety of chemical drugs. For example, self-assembled glycyrrhizic acid-hydroxycamptothecin (GL-HCPT) micelles [121] greatly enhanced HCPT solubility and stability and demonstrated greater antitumour activity and biosafety than that of HCPT injection and HCPT/GL physical combination. By modifying the self-assembled nanopreparation, researchers may achieve the desired properties, such as the integrated function of tumour diagnostics and therapy. Gallic acid (GLA) is a polyphenolic chemical component found in plants, such as rhubarb, Eucalyptus macrophylla (Myrtaceae), and Cornus officinalis (Cornaceae) [122, 123]. An et al. [124, 125] demonstrated that GLA-Fe@BSA-PTX NPs exhibit excellent biological safety and can be utilized for both tumour diagnostics and therapy. The NPs might be utilized for tumour MRI comparison and chemical-photothermal combination therapy, which overcomes the limitations of single therapy and offers tremendous promise in tumour diagnosis and treatment. An irinotecan hydrochloride-curcumin nanosystem (SICN) [16] formed by Cur and irinotecan hydrochloride is a carrier-free SAN. These NPs can synergistically exert the antitumour effect of the two drugs. The NPs exhibit a self-monitor ability due to the fluorescent qualities of Cur. This type of multifunctional carrier-free NP offers a novel approach to tumour combination treatment.

In brief, active components of TCM can self-assemble with chemical drugs to generate nanoformulations. This method frequently outperforms single drug treatment or a simple combination of drugs. It may also be adapted to create a multifunctional nanodiagnostic and therapeutic platform with extensive cancer application potential.

### Other natural self-assembled nanocarriers that deliver active components of TCM

Natural self-assembled nanocarriers (mostly chitosan, liposomes, and so on) can also be used to deliver active ingredients of TCM to CSAN. Given the importance of green environmental preservation and biosafety, the distribution strategy of nonnatural materials is not covered in this study. Employing natural carriers to convey TCM active ingredients can increase TCM active ingredient solubility, bioavailability, and other qualities, as well as improve TCM active ingredient application.

Chitosan is a natural polymer that has been deacetylated from chitin. Chitosan and its derivatives are biocompatible, biodegradable, biologically active, and immunogenic. Hence, the use of chitosan to deliver active components of TCM shows great promise [126–128]. Cur is the primary polyphenolic component in *Curcuma longa*; however, its clinical use is restricted by its poor solubility and bioavailability [129, 130]. Lokesh Pathak et al. [131] employed an ionic gelation technique to prepare biodegradable, biocompatible anionic lipids (soya lecithin) and cationic biopolymers (chitosan) into lecithin/chitosan NPs and entrapped Cur to form Cur NPs, which exhibited antioxidant effects superior to those of free Cur. Artemisinin (AST) is currently the most extensively utilized artemisinin derivative. Renu Chadha et al. [132] loaded AST into lecithin/chitosan NPs composed of chitosan and lecithin to increase its antimalarial efficacy. In P. berghei-infected mice, oral administration of AST NPs resulted in higher antimalarial activity in vivo and a reduction in the parasitism rate. Eman M. El et al. [133] loaded ferulic acid with modified chitosan and conjugated glycyrrhizin to the surface of chitosan NPs. The prepared ferulic acid-conjugated MC NPs showed an excellent ability to target livers. The novel self-assembling bottlebrush polyethylene glycolpolynorbornene-thiocresol block copolymers (PEG-PNB-TC) [134]. Cur and PTX were successfully loaded to form PTX-CUR coloaded PEG-PNB-TC, which shows the potential to treat drug-resistant cervical cancer. Liposomes are bilayer-structured closed vesicles that can be employed as natural nanocarriers to carry TCM active ingredients. When nanoliposomes reach the human body, they are nontoxic, offer biocompatibility benefits, and may be modified to accomplish targeting. Psoralen-loaded nanosized liposomes increase psoralen skin deposition and exhibit high biocompatibility.

### Self-assembled nanoformulations modified by active components of TCM

Some active components of TCM show considerable activity, and nanoformulations xhibit specific activity after CSAN is modified with active components of TCM. Therefore, self-assembled nanoformulations involving the active components of TCM are also included in this paper. Notably, this method coincides with the theory of TCM.

Borneol (Bor) is a monoterpenoid component derived from *Dryobalanops aromatica Gaertn f.* and *Blumea balsamifera DC*. Bor has been widely used in TCM [135, 136] as well as modern medicine; in particular, Bor stimulates the opening of the blood‒brain barrier (BBB) and improve drug penetration and distribution in the brain in a reversible manner, which improve the delivery efficiency of nanomedicines. Guo et al. [137] self-assembled carmustine (CMS) into a micelle and prepared Pep-1/Bor/CMS-M by bifunctionally modifying Bor and Pep-1, which actively target brain glioma cells and hence enhance CMS accumulation in glioma. Lv et al. [138] designed Bor combined with CGKRK peptide-modified PTX prodrug self-assembled redox-responsive NPs (CGKRK-PSNPs), which provides a good effect on the treatment of glioblastoma multiforme (GBM). In TCM, Bor opens the body to awaken the mind and induces drug uptake. This may be related to the use of Bor-modified nanomedicine in CSAN to promote drugs to penetrate the BBB and improve the distribution of drugs in the brain. In clinical practice, Bor is often used as a channel ushering drug, such as in the classical formula compound Danshen dripping pill (CDDP). This is similar to the role of Bor in the modification of CSAN, and the strategy of using some TCM components with special activities for self-assembly is promising.

## Application and advantages of CSAN in cancer

Cancer is a severe public health issue that endangers human life and health. Currently, the primary treatment methods include surgery, radiation, chemotherapy, immunotherapy, and other treatments, among which chemotherapy remains the most common treatment [139–141]. Many chemotherapy drugs, however, are known to induce a variety of adverse effects, and traditional single-agent chemotherapy is known to result in poor therapeutic effects, multidrug resistance, tumour recurrence, and metastasis. Combination chemotherapy combines two or more therapeutic drugs that target separate signalling pathways during tumour growth to achieve synergistic anticancer effects [142, 143].

According to studies, many active components of TCM exhibit strong antitumour effects, high efficiency, low toxicity, multiple actions, various targets, and other benefits and can mitigate the side effects of chemotherapy to some extent [144–146]. In TCM, there are several active components that act on multiple targets simultaneously to produce additive or synergistic effects. TCM's antitumour effect includes increasing the activity of immune cells such as thymus-dependent lymphocytes and natural killer cells to kill tumour cells or directly acting on tumour cells to inhibit tumour growth and metastasis, as well eliminating the side effects or complications of chemotherapy, radiotherapy, or surgical resection [147, 148].

**Fig. 3:**
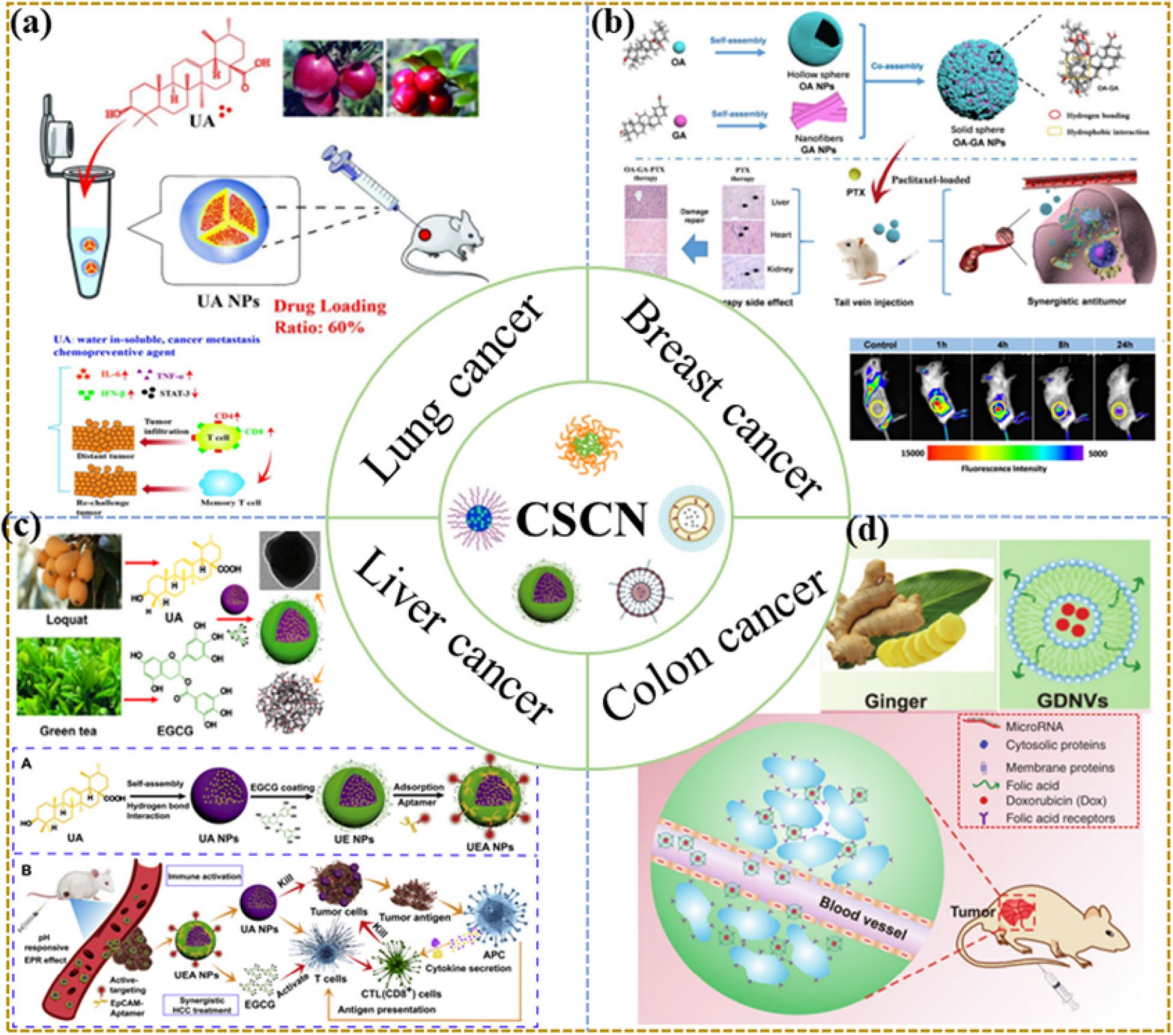
Application of CSAN in different tumors. **a** UA NPs for lung cancer. Copyright 2018 Elsevier. **b** OA-GA-PTX NPs for breast cancer. Copyright 2020 American Chemical Society. **c** UEA NPs for liver cancer. Copyright 2020 Elsevier. **d** DOX-FA-GDNVs for colon cancer. Copyright 2018 The American Society of Gene Therapy

The combination of antitumour active components of TCM and chemotherapeutic drugs for antitumour treatment has attracted increasing attention. To date, combining the active components of TCM with first-line chemotherapy drugs has been shown to be effective at reversing tumour treatment resistance, making the treatment more effective and reducing adverse side effects. A variety of nanoformulations have been used in combination chemotherapy for malignancies to address the inadequacies of free drugs, but nanoformulations with targeting and high safety are still lacking. Self-assembled nanoformulations based on CSAN can improve the solubility and bioavailability of TCM active components as well as tumour targeting. They can also be combined or loaded with chemical drugs with high side effects to play a combined therapeutic role, delay or inhibit the absorption of toxic components, and reduce side effects. The nanoformulations offer significant safety benefits and curative effects as a natural substance (Fig. 3). In vivo safety is critical in the clinical use of nanoformulations, and due to its excellent safety, CSAN will likely be applied in clinics. The application of CSAN in different tumours is summarized in Table 2.

**Table 2:** The application of CSAN in the field of antitumor

Tumor type	Nano preparation	Drug composition	Effects	Characteristic	Refs.
Lung cancer	UA NPs	UA	↓Tumor proliferation and growth ↑Cell apoptosis,↑activation of CD4 + T cells	The ability of liver protection in vivo The potential for immunotherapy	[12]
Lung cancer	PTX-BBR NPs	BBR	↑ROS levels, ↑Block the cell cycle and cell apoptosis ↓Tumor growth	Mitochondria-targeting delivery Synergistic enhancement	[13]
		PTX			
Lung cancer	Rb1/PPD NPs	Rb1	↑Accumulation at the tumor site, ↑Cytotoxicity ↑Blood circulation time, ↓Damage to normal tissues	Synergistic enhancement Simple, scalable, green economy process	[153]
		PPD			
Breast Cancer	OA-GA-PTX NPs	OA	↑Drug releasing capacity and Cellular uptake ↑Tumor aggregation targeting and apoptosis ↓Tumor proliferation and growth, ↑Block the cell cycle ↓ALT、AST、LDH、CK、MDA, ↑SOD、GSH	Synergistic enhancement Reduce chemotherapy side-effects Different antitumor mechanisms Reduce damage caused by oxidative stress	[29]
		GA			
		PTX			
Breast Cancer	UA-PTX NPs	UA	↓Tumor growth ↑Block the cell cycle, ↑Cell apoptosis and cellular uptake ↑Tumor aggregation targeting, ↑SOD、GSH	Synergistic enhancement Different antitumor mechanisms Reduce chemotherapy side-effects	[19]
		PTX			
Breast Cancer	DTX/GBA-PLGA NPs	GA	↑The expression of P-gp, ↑Cell apoptosis ↓Tumor growth	Synergistic enhancement Special in multidrug-resistant breast cancer	[81]
		DTX			
Liver cancer	UEA NPs	UA	↑Drug releasing capacity and cellular uptake, ↓Tumor growth, ↑IL-12、IFN-γ、CD4^+^、CD8^+^ ↑Tumor aggregation targeting, ↑Cytotoxicity	Synergistic enhancement Improvement of Th1/Th2 imbalance	[102]
		EGCG			
		DOX			
Liver cancer	GL-HCPT micelles	GL	↑Cytotoxicity, ↑Tumor aggregation targeting ↓Tumor growth	Improve the solubility and stability of HCPT Reducing the side effects of HCPT	[121]
		HCPT			
Gastric cancer	SiCN	Cur	↑Drug releasing capacity and cellular uptake ↑Cytotoxicity, ↑Endocytosis	Synergistic enhancement Self-monitoring function	[16]
		Irinotecan hydrochloride			
Colorectal cancer	DOX-FA-GDNVs	GDNV	↓Tumor proliferation and growth, ↑Cell apoptosis ↑Drug releasing capacity and targeting ability	Loading Dox with high efficiency	[92]
		DOX			
Colorectal cancer	INP-DOX	INP	↑Cytotoxicity, ↓Tumor growth	Synergistic enhancement PH-responsive	[74]
		DOX			
Ovarian cancer	PTX-ss-TMP NPs	TMP	↑Cytotoxicity and cell apoptosis, ↑Block the cell cycle ↑Drug releasing capacity and cellular uptake ↑Tumor aggregation targeting, ↓p-VEGFR2/VEGFR2, p-AKT/AKT, p-mTOR/mTOR and p-p38/p38	Synergistic enhancement Redox-responsive drug release Anti-angiogenesis effect	[118]
		PTX			
Cervical cancer	PTX-Cur coloaded PEG-PNB-TC	Cur	↑Endocytosis and cellular uptake, ↑Cytotoxicity	Circumvented the PTX resistance in the MDR cancer cells	[167]
		PTX			
Glioma	Pep-1/Bor/CMS-M	Bor	↑Cytotoxicity and cell apoptosis, ↑Endocytosis and cellular uptake, ↓Tumor growth	Blood–brain barrier penetrating	[137]
		CMS			
Neuroglioma	Lut-Fe3^+^ NPs	Lut	↓Tumor proliferation, ↑Cytotoxicity	High oxidation stability Chemotherapy combined with PPT	[15]
Melanoma	OPDMA-Cela NPs	Cela	↑Pharmacokinetics, ↑Mitochondria targeting capacity ↓Tumor growth	Mitochondria-targeted carrier for drug delivery	[41]

### Lung cancer

Lung cancer has been the most common type of malignant tumour in China over the last 30 years [149–151]. Many studies have found that numerous active components of TCM, such as UA, BBR, and ginsenoside, exhibit an intervention effect on lung cancer, and their stability and therapeutic efficacy were improved when they were self-assembled to form NPs.

UA NPs [12] can significantly inhibit proliferation and induce apoptosis in A549 human lung adenocarcinoma cells, reduce the expression of COX-2/VEGFR2/VEGFA, increase the immunostimulatory activities of TNF-, IL-6, and IFN-, and decrease STAT-3 activity, which can significantly improve CD4 + T-cell activation. UA NPs have the ability to suppress tumours, protect the liver, and provide immunotherapy. PTX-ss-BBR NPs [13] can actively target mitochondria, boost ROS levels in A549 cells, block A549 cells in the G2/M stage, induce apoptosis in A549 cells, and suppress tumour development. PTX-ss-BBR NPs were more effective against *Staphylococcus aureus*, which was linked to lung cancer incidence, indicating their potential for treating bacterially-induced lung cancer. The high hydrophobicity of PTX significantly reduced its bioavailability. This limitation might be addressed by self-assembled nanoformulations. Han et al. [152] synthesized PTX-ss-PTX and loaded it with 1,1-dioctadecyl-3,3,3,3-tetramethylindotricarbocyanine iodide (DiR) to form DiR-loaded self-assembled NPs (DSNs). The disulfide bond in DSNs was exploited as a redox response bond to promote the rapid release of PTX in tumour cells. In in vivo and in vitro studies, DSNs demonstrated strong antitumour effects in chemotherapy and photothermal combination treatments. Protopanaxadiol (PPD-type) is the most important active component in *Panax ginseng* (Araliaceae). In in vivo and in vitro studies, Rb1/PPD NPs [153] exhibited the EPR effect of NPs and the synergistic effect of Rb1/PPD, resulting in a superior anti-lung cancer effect and adequate safety. Simultaneously, Rb1 NPs can be employed to distribute insoluble drugs, decrease adverse effects, and enhance anticancer benefits.

### Breast cancer

Breast cancer is among the most frequent malignant tumours in women, with the highest incidence rate among malignant tumours in females [154–156]. The active components of TCM in the treatment of breast cancer are continually being investigated. CSAN has the potential to boost the activity of TCM for the treatment of breast cancer, exert a synergistic effect in chemotherapy with the self-assembly of chemotherapy drugs, and lessen the side effects of chemotherapy drugs, such as PTX and DOX. It can also self-assemble with Fe3^+^, allowing nuclear magnetic resonance imaging as well as photothermal treatment to be performed with NPs.

Both in vivo and in vitro, OA-GA-PTX NPs [29] demonstrated powerful antitumour effects, achieving a maximum inhibitory rate of 82.6% for 4T1 breast cancer tumour-bearing mice. Additionally, the NPs may upregulate key antioxidant pathways to prevent PTX-induced liver damage. LDA exhibited an ability to self-assemble into LDA NPG and can be utilized to load DOX, resulting in DOX-LDA/NPG [14], which showed a synergistic anti-breast cancer effect and reduced DOX-induced cardiotoxicity. The UA-PTX NPs [19] preserved the antitumour and liver-protective properties of UA. Furthermore, these NPs demonstrated a synergistic effect in inhibiting tumours both in vivo and in vitro. Additionally, they had the ability to reduce liver damage caused by PTX. Another promising option is DTX-loaded BSP-SA NPs [81], which exhibit a strong drug release effect and are safe for use. Studies have shown that these NPs exhibit greater efficacy in combating cancer than that of free DTX while also minimizing the toxicity of DOX in the heart and kidneys. Wang et al. [157] developed 2-glucosamine-fluorescein-5(6)-isothiocyanate-glutamic acid-paclitaxel (2DA-FITC-PTX NPs) through self-assembly, which demonstrated a notable enhancement in tumour targeting and growth suppression in breast cancer cells. GLA-Fe@BSA-PTX NPs [125] were found to possess several desirable characteristics, including a moderate size, excellent water dispersibility and stability, low cytotoxicity, strong magnetic resonance imaging contrast, and the ability to provide combination chemo-photothermal treatment for patients with breast cancer. DTX/GBA-PLGA NPs [120] were found to induce increased apoptosis of MCF-7/ADR cells by downregulating the expression level of P-gp. *T*he most effective inhibition of tumour growth was observed in in vivo studies. The synergistic antitumour activity produced by GBA-PLGA NPs showed significant potential in the treatment of multidrug-resistant breast cancer.

### Liver cancer

Liver cancer is a highly common form of malignancy characterized by a low survival rate and high mortality rate among patients [158–160]. However, TCM has been found to contain active components that are effective in the treatment of liver cancer. CSAN exhibits a better therapeutic effect on liver cancer due to self-assembly of antitumour components of TCM or coassembly with chemotherapy drugs, and it can be actively or passively targeted to improve the drug delivery ability of nanoformulations.

UEA NPs [102] showed the ability to target EpCAM-positive liver cancer cells, exhibited strong cytotoxicity against HepG2 cancer cells, had higher tumour inhibition rates in vivo, and can provide large synergistic therapeutic benefits via immunotherapy that stimulates innate and acquired immunity. DOX/ASP-DOCA NPs [84] reduced cell growth by being preferentially internalized into HepG2 cells via ASPR recognition. DOX/ASP-DOCA NPs, on the other hand, can precisely target HepG2 solid tumours, leading to improved anticancer efficacy. HCPT, derived from *Camptotheca acuminate* (Nyssaceae), is a typical DNA topoisomerase I (Topo I) inhibitor with potent anticancer activity. However, HCPT is poorly soluble and unstable. GL-HCPT micelles [121] can greatly increase HCPT solubility and trigger apoptosis in HepG2 and Huh7 human hepatoma cells. In vivo studies revealed that compared to hydroxycamptothecin injection, GL-HCPT micelles achieved superior anticancer efficacy and safety. CP3-DOX NPs [70] can accelerate DOX release in the acidic pH of the tumour microenvironment, exhibit stronger cytotoxicity to HepG2 cells than that of free DOX, and promote early cell apoptosis.

### Other tumours

CSAN has also been used to study different types of cancer. Because of its superior stability and biocompatibility, stronger synergistic therapeutic impact, and improved safety, its research possibilities in the field of malignancies are promising.

Gastric cancer is among the most frequent types of cancer that endangers human health. The rate of early detection is low, and few tumour indicators are available [161, 162]. Through combination therapy, CSAN can provide a greater anticancer impact. SiCN [16] exerted the synergistic therapeutic effect of irinotecan hydrochloride and Cur, had greater cytotoxicity, induced apoptosis and necrosis of HGC-27 human gastric cancer cells, arrested the cell cycle, and could monitor its uptake, absorption, and excretion in real time in vivo and in vitro.

Colorectal cancer is a common malignant tumour of the digestive tract [163, 164]. CSAN can deliver DOX through tumour targeting and provides a better anticancer effect. Ginger-derived GDNV [92] can be efficiently absorbed by colon cancer cells and loaded with DOS to treat colon cancer. Dox-FA-GDNVs can better cause apoptosis in colon-26 cells. In vitro and in vivo studies showed that compared to free Dox, they were more effective and safer. INP-DOX nanoconjugates [74] exhibited a much higher cytotoxic activity than that of free DOX on a variety of cancer cell lines. The tumour volume of resistant SW620/Ad-300 colon cancer cells was considerably lower in the INP-DOX group than in the free DOX group.

Ovarian cancer exhibits invasion and metastatic features with a very high fatality rate, and global morbidity and mortality are rising yearly [165, 166]. Several active components of TCM have been identified as having substantial anti-ovarian cancer action. CSAN combine the anti-ovarian cancer active components of TCM with PTX to create a therapeutic synergy. PTX-ss-TMP NPs [118] exhibited a good synergistic therapeutic effect on ovarian cancer. In vitro and in vivo studies revealed that PTX-ss-TMP NPs played a combined role in inhibiting the proliferation of ovarian cancer cells and angiogenesis and effectively inhibited the progression of tumours. TF-targeted PTX and CUR coloaded micelles [167] can better infiltrate NCI-ADR-RES ovarian cancer NCI-ADR-RES cell spheroids, and they have shown strong antiovarian cancer action in vivo.

Cervical cancer is the leading cause of cancer mortality among women in developing countries [168, 169]. Cisplatin (DDP) is the first-line treatment indicated for advanced, recurring, or metastatic cervical cancer. The molecular mechanism of Cur-induced cervical cancer cytotoxicity involves several targets, and the codelivery of DDP and Cur to tumour tissues is appealing. DDP and Cur-loaded lipid-polymer hybrid NPs (D/C/LPNs) [170] outperformed other preparations in terms of anti-cervical cancer tumour activity in vivo and in vitro. The synergistic anti-cervical cancer impact of PTX-Cur coloaded PEG-PNB-TC [134] was superior to that of the other treatment groups, and it could effectively overcome PTX resistance in MDR cancer cells.

Glioma is the most common primary intracranial malignancy and is usually invasive, has no apparent borders and is difficult to entirely resect with standard surgery [171]. Poor BBB penetration and limited accumulation of therapeutic drugs at tumour locations are significant barriers to glioma therapy. Pep-1/Bor/CMS-M [137] may target gliomas with IL-13 receptor overexpression, cross the physiological barrier associated with brain microvascular endothelial cells, considerably boost human glioma BT325 cellular cytotoxicity, significantly improve internalization, and effectively induce cell apoptosis. In the treatment of orthotopic glioma-bearing nude mice, Pep-1/Bor/CMS-M showed the strongest tumour growth inhibition, the longest life duration, and the lowest systemic toxicity. CGKRK-PS NPs [138] can significantly improve anti-glioma activity and increase median survival time (39 days). LuT/Fe3^+^ NPs [15] exhibited photothermal and chemotherapeutic effects. It can raise the temperature by more than 20 °C in less than 10 min and generate a photothermal conversion efficiency of 26.0%. LuT/Fe3^+^ NPs exhibited greater cytotoxicity to U87MG glioma cells than that of Lut alone.

Cela, a natural triterpene component found in TCM, has been found to be useful in the treatment of melanoma [172], but its poor water solubility, short plasma half-life, and significant systemic toxicity have limited its clinical use. OPDMA-Cela NPs [41] exhibit better pharmacokinetics and mitochondrial targeting capacity and can cause considerable immunogenic cell death, improve Clea's therapeutic effect in vivo, and reduce systemic toxicity.

By using the EPR effect of NPs and the active targeting that can be achieved through modification, CSAN may overcome the problems of poor solubility and low bioavailability of antitumour active ingredients and better target tumour cells. Self-assembly exert the active component's antitumour effect and be combined with other antitumour drugs to exert a combined chemotherapeutic effect, as well as reduce toxic and side effects. Nanoformulations can be become photothermal or magnetic by modification, allowing for the integration of tumour diagnostics and therapy. Due to the advantages of advantages self-assembled nanopreparations of TCM, including high efficiency, plasticity, and safety, these nanopreparations show great potential in tumour diagnosis and treatment.

## Conclusions

CSAN employs the self-assembly properties of TCM's natural activity to self-assemble a drug or combine it with other drugs to generate nanopreparations. The preparation process is straightforward and ecologically friendly, which can increase drug solubility and bioavailability while also increasing delivery efficiency via NP targeting. At the same time, CSAN is very safe and can lessen the side effects of chemotherapy drugs. The research conducted by CSAN holds great significance in enhancing the potential application of TCM, studying TCM theory, developing new TCM medications, and promoting the clinical transformation of nanoformulations. This research is expected to play a crucial role in modernizing TCM.

### Exploiting the application potential of TCM

TCM has a lengthy history of usage and significantly contributed to the health of people in Asian. TCM's natural active components are secondary metabolites with low molecular weights that are derived naturally from plants, animals, or minerals and display a variety of pharmacological effects, high biocompatibility, and regulated degradation [173]. These active components involve drawbacks, such as poor stability, a short half-life in vivo, a rapid clearance rate, a modest curative impact, and poor water solubility; however, they can be self-assembled into stable and beneficial nanoformulations. Simultaneously, due to its outstanding absorptivity, biodegradability, biocompatibility, and safety, SAN is a novel form of natural drug delivery nanocarrier that can replace artificially manufactured nanomaterials; in addition, SAN may be utilized to transport drugs to improve efficacy and decrease side effects. The construction of self-assembled nanosystems of natural TCM active components can fully exploit their self-assembly capabilities and enable the development of novel TCM active component applications.

### Studying TCM theory from a new perspective

In clinical practice, it is essential to use medicine under the guidance of TCM theory. The development of CSAN is closely linked to TCM theory and offers a fresh perspective for its study. TCM's compatibility theory suggests that certain drug pairs can have a synergistic effect, such as Rhubarb and Coptis, Scutellaria and Coptis. This theory can also reduce the toxicity of drug pairs. For example, AA-containing herbs and BBR-containing herbs can reduce toxicity when used in combination, which is also similar in the self-assembly products between them. Research on CSAN may provide a new perspective for revealing the synergistic and attenuating effect of TCM compatibility theory [101]. The CSAN, which is guided by TCM theory, has the potential to expand the clinical use of many Chinese medicinal plants. At the same time, it has been found that the SAN present in the decoction is closely linked to its curative effect. This approach of exploring the onset mechanism of TCM decoction through the perspective of SAN is innovative. However, research on CSAN is currently not extensive enough. Therefore, CSAN needs to be further investigated under the guidance of TCM theory, and this direction requires more effort in the future.

### Developing novel TCM medicines

TCM is a fantastic treasure trove. Many freshly licenced small molecule novel drugs are now generated from natural phytochemicals [174]. In clinical practice, TCM compound decoction is a frequent medicinal dosing type. However, the constituents of TCM compounds are intricate, their mechanisms of action are often unknown, and performing quality control is challenging. Effective isolated during separation and purification cannot adequately reflect the components present in the compounds and explain the onset mechanism of the components. SAN may exhibit greater biological activity than that of extracted active components or simple combinations of active ingredients. SAN might be the true form of the material basis for the effectiveness of TCM and compound prescriptions. Some SANs have been shown to improve the absorption of active ingredients in TCM. Investigating the causes behind SAN's increased efficacy can aid in the development of new and improved Chinese medicines. SAN might be a new research strategy for developing novel TCM medications.

### Promoting the clinical transformation of nanoformulations

Nanoformulations have tremendous benefits over traditional drug preparations and are frequently employed to deliver insoluble drugs, antitumour drugs, gene drugs, and drugs that must cross the blood‒brain barrier. Basic research on nanoformulations is active, but few breakthroughs have been achieved in clinical transformation. Safety concerns and problems in the preparation process are the primary reasons that restrict the clinical transformation of nanoformulations. The Food and Drug Administration (FDA) has now authorized a few nanoformulations and demands that nanotherapeutic preparations are totally removed in the body within a reasonable period. In terms of safety, CSAN provides benefits that traditional nanomedicines lack, such as the ability to cooperate with pharmaceuticals in addition to exhibiting particular biological properties. Simultaneously, the preparation method is often easy to perform, ecologically benign, and requires no expensive equipment or precious ingredients. CSAN is predicted to contribute to the clinical transformation of nanoformulations due to these properties.

There are certain issues with current CSAN research. The constituents of self-assembled drugs restrict the therapeutic efficacy of SAN, and present research is primarily limited to single-drug research or two-drug combinations. There have been a few reports of three-drug or even multidrug self-assembly, whereas multidrug use is common in clinics. Nanostructure construction is strongly reliant on molecular configuration, intermolecular forces, and spatial molecular organization, making its study reliant upon chance. The formation force of CSAN is mostly the weak interaction of molecules, which exhibits low physical and chemical stability. Improving SAN stability will be a critical challenge in the future. To date, only a few studies have been conducted to investigate the effectiveness mechanism and safety of SAN. SAN is more effective than active components or their basic mixture. However, the precise method through which SAN promotes the absorption of active components and exerts more biological activity is unknown. Biotransport characteristics such as SAN absorption, distribution, and degradation in vivo, as well as cellular uptake and transport mechanisms, must be investigated further. Furthermore, the toxicity of several active substances in TCM should be evaluated more comprehensively and thoroughly. SAN research is now applied to only a few kinds of illness, and this area must be extended in the future.

The self-assembly of TCM natural products is a key step in the development of TCM natural products, and the potential of CSAN will be further explored as research progresses. The modernization of TCM necessitates cross-disciplinary collaboration and innovation, encouraging the integration of TCM with modern scientific and technological methods. While preserving the fundamental principles of TCM theory, it is crucial to remain innovative and progressive to revitalize the practice. Self-assembly technology utilized by the CSAN presents an opportunity to explore the application of TCM, and the integration of TCM and self-assembly nanotechnology can offer innovative strategies and viewpoints to advance the modernization of TCM. In conclusion, CSAN shows significant research value and potential and should be further studied and explored. The anticipated role of CSAN in modernizing TCM and contributing to human health is important and promising.

## Data Availability

Not applicable.

## Abbreviations

TCM: Traditional Chinese Medicine
CSAN: Chinese medicine self-assembly nanostrategy
NPs: Nanoparticles
SAN: Self-assembly NPs
BBR: Berberine
CA: Cinnamic acid
Rhe: Rhein
3,4, 5-Tca: 3,4, 5-Methoxyic acid
Sito: B-sitosterol
OA: Oleanolic acid
GA: Glycyrrhetinic acid
UA: Ursolic acid
PTX: Paclitaxel
Cela: Celastrol
BA: Baicalin
WOG: Wogonoside
NFs: Nanofibers
BDS: BBR derivatives
RHL: Rhamnolipids
LDA: Liquidambaric acid
Lut: Luteolin
DOX: Doxorubicin
POD: Podophyllotoxin
Pae: Paeoniflorin
NPG: Natural product gels
GLP: Glycated Licorice Protein
RPP: Radix Pseudostellariae Protein
Cur: Curcumin
SAP: Semen Armeniacae Amarum Protein
BLG: Ban-Lan-Gen decoction
SA: Stearic acid
ASP: Angelica polysaccharide
GDNVs: Ginger-derived nanovectors
GDLVs: Ginger derived lipid vectors
MRSA: Multidrug-resistant Staphylococcus aureus
AC: Aconitine
AA: Aristolochic acid
AKI: Acute kidney injury
GW: Genkwanine
EGCG: Epigallocatechin gallate
EpCAM: Epithelial cell adhesion molecule
UEA NPs: UA@EGCG-Apt NPs
CCD: Coptis chinensis decoction
TJs: Tight junctions
GQD: Ge-Gen-Qin-LianTang decoction
MXSGT: Ma-XingShi-Gan-Tang decoction
TMP: Tetramethylpyrazine
GBA: Gambogic acid
GLA: Gallic acid
AST: Artemisinin
Bor: Borneol
BBB: Blood–brain barrier
CMS: Carmustine
GBM: Glioblastoma multiforme
CDDP: Compound Danshen dripping pill
DiR: 1,1-Dioctadecyl-3,3,3,3-tetramethylindotricarbocyanine iodide
Topo I: Typical DNA topoisomerase I
FDA: Food and Drug Administration
